# Shaping the Future: The Transformative Path of the Arab Board of Rheumatology

**DOI:** 10.7759/cureus.45624

**Published:** 2023-09-20

**Authors:** Khalid A Alnaqbi, Salwa A Al Cheikh

**Affiliations:** 1 Rheumatology Division, Tawam Hospital, Al Ain, ARE; 2 Internal Medicine Department, College of Medicine and Health Sciences, UAE University, Al Ain, ARE; 3 Internal Medicine, Damascus University, Damascus, SYR; 4 Rheumatology Department, Al Assad University Hospital, Damascus, SYR

**Keywords:** accreditation council for graduate medical education (acgme), curriculum, medical education, manpower, health specialization, fellowship program, rheumatology

## Abstract

Rheumatology fellowship programs represent a pivotal juncture for aspiring specialists, embarking on a transformative journey of expertise and care. The League of Arab States, comprising 22 nations and a collective population of 464.68 million as of 2022, established the Arab Board of Health Specialization in February 1978. This visionary initiative aimed to curb the emigration of Arab physicians and address the scarcity of specialized medical practitioners in the Arab world. Since the establishment of the Internal Medicine specialty in 1979, the curriculum and examinations have undergone sustained refinement and enhancement. In a significant stride, the Arab Board established the Scientific Committee of the Rheumatology Fellowship Program on November 28, 2022. Its main goal is to ensure that graduating fellows will be of high caliber and can contribute to the care of patients with rheumatic disease in the Arab world. This editorial illustrates the historical trajectory of the Arab Board's evolution and chronicles the dynamic expedition of shaping the rheumatology fellowship program.

## Editorial

Introduction

Rheumatology fellowship programs mark the beginning of a transformative journey for aspiring specialists. These programs provide a structured pathway for medical graduates to delve into the intricate realm of rheumatic diseases. Through a blend of comprehensive clinical exposure, in-depth research, and rigorous training, fellows acquire the expertise needed to diagnose and manage a wide spectrum of musculoskeletal and autoimmune conditions. These initial steps in rheumatology fellowships lay the foundation for a career dedicated to improving the lives of patients suffering from a variety of challenging musculoskeletal and autoimmune diseases.

The League of Arab States comprises 22 countries with a total population of 464.68 million as of 2022 [1]. Arab countries span North Africa, Western Asia, and a segment of East Africa that share a rich cultural heritage, and each holds its own unique traditions, histories, and landscapes. The official language is Arabic, in addition to other languages in some countries, such as English, French, Amazigh, Somali, and Comorian. The inhabitants of Arab countries come from diverse ethnic backgrounds such as Arabs, Kurds, Amazigh, Somalis, Assyrians, Armenians, and Circassians [2].

This article will address the history of the Arab Board of Health Specialization with a view to shed some light on the recent birth of the Arab Board of Rheumatology.

The Arab Board of Health Specialization

Arab Board of Health Specialization (or simply the Arab Board) is an independent regional organization that provides postgraduate medical education and training programs across various health specialties. It was established in February 1978 in Kuwait by the Arab League’s Council, which encompasses the Ministries of Health of the Arab countries. The General Secretariat is located in Damascus, Syria. The organization's original name was the Arab Board of Medical Specialization. However, in March 2009, the name was changed to the Arab Board of Health Specialization to reflect the decision to allow the inclusion of trainees from different health specialties. The idea was conceptualized as a solution to reduce the emigration of Arab physicians to non-Arab countries, and to address the scarcity of specialized physicians [3].

The Arab Board plays a pivotal role in ensuring the quality and standardization of medical education and training across its member countries. It establishes guidelines, curricula, and assessment methods to maintain a consistent level of competence among specialists. The Arab Board currently offers 60 programs with many subspecialties in a wide range of medical specialties, including internal medicine, dermatology, ophthalmology, general surgery, pediatrics, obstetrics and gynecology, radiology, anesthesia, psychiatry, and community medicine. The members of the Scientific Committees for any specialty within the Arab Board are nominated by the ministries of health of the Arab countries.

Notably, the curricula of many of these programs are adopted from various international specialization programs. Moreover, many board members from these specialties have received their education from Western countries. Overall, these programs provide structured training and education to physicians who have completed their basic medical degrees. The training programs typically involve a combination of supervised clinical training, rotations, theoretical education, and assessments. Trainees are required to complete a certain number of years of training and meet specific competency requirements. The training culminates in a series of examinations, including written and oral assessments, prepared and rigorously revised by the Examination Committee.

An Examination Committee is made up of expert physicians in medical education. It prepares written and practical [objective structured clinical examination (OSCE)] questions, which are vetted and revised regularly. The Arab Board conducts exit (written and practical) examinations regularly throughout the year, which are held in different Arab countries. Upon successful completion of the Arab Board's training and examination requirements, physicians are awarded certification in their chosen health specialty. This certification is recognized by participating Arab countries and institutions.

For a local specialty program to receive the Arab Board accreditation, an application from the requesting healthcare facility must be completed, followed by a routine visit from the Arab Board staff to that facility.

The Arab Board collaborates with medical institutions, universities, and ministries of health within member countries to facilitate the implementation of its training programs. It also works to ensure that the training provided aligns with international standards and fosters international collaboration to support the goals of the Arab Board and the accreditation of its programs and certifications.

The timely need for the Arab Board of Rheumatology

On January 10, 1979, the Internal Medicine program of the Arab Board was established. Throughout the years, its curriculum and examinations have undergone continuous development and improvements. As of August 2023, 2,975 physicians have been certified by the Arab Board in the specialty of Internal Medicine. 

Some Arabic countries have established local rheumatology specialization programs, while others do not have the expertise or manpower to establish such programs that would provide an opportunity for interested physicians to specialize in rheumatology. Such countries have a shortage of rheumatologists, which negatively affects the care of patients with rheumatic diseases who are instead managed by general internists or orthopedic surgeons. In order to increase the supply of qualified rheumatologists in these countries, exchange programs with other Arabic and non-Arabic countries have been instituted. 

Table 1 presents a list of Arab countries that have local rheumatology specialization programs. Some Arab countries (such as Syria, Egypt, Algeria, Tunisia, and Morocco) have different pathways for rheumatology specialization programs. For instance, in Syria, Tunisia, Algeria, and Morocco, physicians may begin their specialization in rheumatology after a period of internship, which requires training in internal medicine as part of the rheumatology program. Additionally, certain programs in Egypt integrate rheumatology training with rehabilitation, thereby eliminating the requirement for prior training in an internal medicine program. Conversely, another pathway in Egypt necessitates the completion of an internal medicine training program before specializing in rheumatology.

**Table 1:** Arab countries that have local rheumatology fellowship programs ACGME: Accreditation Council for Graduate Medical Education; CBAHI: Saudi Central Board for Accreditation of Healthcare Institutions; KSA: Kingdom of Saudi Arabia; NIHS: National Institute for Health Specialties; UAE: United Arab Emirates

	Rheumatology specialization program	Completion of internal medicine residency as a prerequisite	Accrediting body	Exit examination
Syria	Yes	No	Syrian Board of Medical Specialties	Yes
Jordan	Yes	Yes	Jordanian Medical Council	Yes
Iraq	Yes	Yes	Medical Education Council in Iraq	Yes
UAE	Yes	Yes	ACGME, NIHS, Saudi CBAHI	Yes
Qatar	Yes	Yes	ACGME	No
KSA	Yes	Yes	Saudi CBAHI	Yes
Egypt	Yes	Depends on the selected pathway	University/teaching hospitals	Yes
Algeria	Yes	No	Ministry of Higher Education	Yes
Sudan	Yes	Yes	Sudan Medical Specialization Board	Yes
Tunisia	Yes	No	University hospitals	Yes
Morocco	Yes	No	University Hospitals accredited by the Ministry of Higher Education	Yes

Furthermore, in the UAE, there are currently two rheumatology fellowship programs: in Abu Dhabi and Dubai. Sheikh Shakhbout Medical City (SSMC) program in Abu Dhabi has been accredited by the Accreditation Council for Graduate Medical Education (ACGME) with no current exit examination. The SSMC program is currently undergoing accreditation by the National Institute for Health Specialties (NIHS), which will include an NIHS-certified exit examination. In early 2023, the Dubai Academic Health Corporation's rheumatology program was accredited by the Saudi Central Board for Accreditation of Healthcare Institutions (CBAHI). The program relies on the Saudi Board for its exit examination and spans a two-year training period.

To the best of our knowledge, countries that do not have local rheumatology fellowship programs are as follows: Kuwait, Oman, Bahrain, Libya, Lebanon, Comoros Islands, Palestine, Yemen, Mauritania, Somalia, and Djibouti.

Establishment of the Arab Board of Rheumatology Fellowship Program

**Figure 1:**
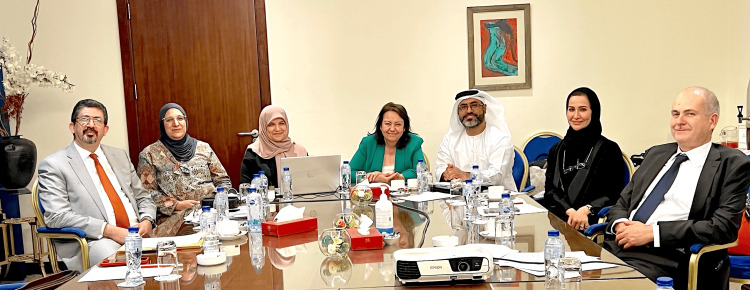
The Scientific Committee of the Arab Board of Rheumatology

Due to the low number of accredited rheumatology specialization programs in the Arab world, and the fact that many physicians desire to receive their training via local fellowship programs, the Arab Board met on October 25, 2015, and approved the idea of establishing the Arab Board of Rheumatology program as a subspecialty program of Internal Medicine specialty. On November 28, 2022, the Arab Board formed the Scientific Committee of the Rheumatology Fellowship Program, represented by Arab countries including Syria, Iraq, Jordan, the UAE, Qatar, Algeria, and Libya (Figure 1).

Adult Rheumatology Fellowship curriculum

The Scientific Committee of the Arab Board Rheumatology Fellowship Program held its first virtual meeting on December 16, 2022. A second face-to-face meeting was held in Amman, Jordan from June 8-11, 2023, where the rheumatology curriculum was established, which was adopted from the ACGME curriculum. Table 2 shows some of the highlights of the curriculum.

**Table 2:** Highlights of the curriculum and examination of the Arab Board of Rheumatology Program ABHS: Arab Board of Health Specialization; DMARDs: disease-modifying anti-rheumatic drugs; NSAIDs: non-steroidal anti-inflammatory drugs

Highlight	Details
Objectives of the program	Set high standards, ensuring that graduates can proficiently care for patients with rheumatic diseases in the Arab world, trainees are prepared for continuous medical education and are well-versed in reading and conducting clinical research
Eligibility criteria for program entry	1) Certification in internal medicine from the ABHS or other certifying bodies recognized by the ABHS, 2) completion of the internal medicine residency program, and 3) the number of trainees is determined by the local rheumatology program requirements
Duration of the program	Two years after completing internal medicine training. If the duration of the local rheumatology program is 3 years, then trainees are not eligible for the Arab Board examination until the completion of the third year
Measurable skills	1) Patient care, 2) medical knowledge, 3) system-based learning, 4) communication skills, 5) professionalism, and 6) practice-based learning
Criteria for accrediting training hospitals for rheumatology	1) Accreditation of an internal medicine residency program by the ABHS, 2) internal medicine residents have graduated from the requesting hospital’s program, 3) the hospital has an accredited internal medicine program and is affiliated with orthopedic surgery, obstetrics and gynecology, pediatrics and rehabilitation, and physiotherapy services. 4) the minimum number of rheumatology faculty is 2, and the maximum number of trainees is 3-4 for each year of training, 5) the minimum number of hospital beds is 200, and it is preferable that rheumatology service has a department, 6) availability of rheumatology clinics, 7) inpatient rounds with trainees, 8) weekly educational meetings (e.g., theoretical session, workshops, journal club, clinicopathological session, morbidity and mortality session), 9) availability of a laboratory that offers tests for basic chemistry and hematology, tests for infections, coagulation tests, and tests for autoimmune rheumatic diseases, 10) availability of certain tests for radiology (e.g., X-rays of the joints, CT scan, MRI, musculoskeletal ultrasound, and bone densitometry), 11) availability of pathology service, 12) availability of libraries such as electronic Library, and 13) availability of archives or transcription department
Methods of training	E.g., daily clinical practice, self-learning, didactic teaching sessions, workshops, journal clubs, conferences, and morbidity and mortality sessions
Graded responsibilities of the trainees	Based on history taking, the performance of physical examination, generation of appropriate differential diagnosis, the performance of aspiration (joints, tendons, and bursae), making decisions on the appropriate treatment for patients (including DMARDs, glucocorticosteroids, and NSAIDs), and acquisition of skills to conduct research
Logbook	Consultations and their respective diagnoses, procedures [Injection and/or aspiration with or without ultrasound (of joints, tendons, and bursae, and musculoskeletal ultrasound), interpretation of dual-energy X-ray absorptiometry (DEXA) and X-rays], scholarly activities (e.g., attendance in teaching sessions, conferences, participation in teaching undergraduate students, and conducting research), and medical ethics and professionalism (e.g., attendance, on-calls, leaves, and working hours)
Trainee’s responsibilities	E.g., documentation in medical records, attending inpatient rounds and rheumatology clinics under supervision, commitment to working hours and on-calls, leaves, participation in educational sessions and conferences, engagement in research, and expected ethical commitments
Elective rotations in other Arab countries	A structured program or rotation related to rheumatic diseases is recommended
Final examination	Annual examination comprising a written part (100 multiple-choice questions) and objective structured clinical examination (OSCE) including history taking, clinical examination, communication skills, test interpretation, and miscellaneous stations. The minimum passing score is 70%. A question bank is being developed to provide comprehensive assessment for trainees

More details on the rheumatology program are available on the Arab Board website [4].

Pediatric Rheumatology fellowship

The Scientific Committee has recognized the importance of establishing a pediatric rheumatology training program. Due to the shortage of pediatric rheumatologists in the Arab world, a discussion about combining pediatric rheumatology with adult rheumatology fellowship programs will be conducted in future board meetings.

Conclusion

The Arab Board of Rheumatology Fellowship program has finally been constituted. Its main goal is to ensure that graduating fellows will be of high caliber and can contribute to the care of patients with rheumatic disease in the Arab world. Hospitals and fellowship program staff need to build the basic infrastructure to support rheumatology programs in order to provide an opportunity for trainees to gain expertise in diagnosing and managing a variety of cases in different settings (e.g., outpatient, inpatient, and daycare). Rheumatology fellowship programs across Arab countries can now apply for accreditation from the Arab Board of Health Specialization.
